# Need for and Key Outcomes of a Community-based Frailty Prevention Program: The Busan Health UP Program and Operational Experience in Haeundae-gu

**DOI:** 10.56786/PHWR.2026.19.41suppl.4

**Published:** 2026-10-29

**Authors:** Yeohyun Ahn

**Affiliations:** Haeundae-gu Public Health Center Busan Korea

**Keywords:** Frailty, Health UP Program, Community Health Centers, Community participation

## Abstract

**Objectives**: This article aims to describe the structure and implementation of the Health UP Program, a community-based frailty prevention initiative launched in Busan in 2024 through Community Health Centers, and to examine its implementation, operational experience, and outcomes at four centers in Haeundae-gu. It also seeks to identify future directions and policy implications for community-based frailty prevention.

**Methods**: The Health UP Program framework, launched in 2024, was reviewed, focusing on early frailty screening; multidomain interventions including exercise, nutrition, cognition, and social participation; and a resident-participatory ongoing management structure linking Community Health Groups, Community Health Leaders, and HAHA Health Partners. Pre- and post-program assessment data from 139 participants in Haeundae-gu were analyzed using a two-sided Wilcoxon signed-rank test.

**Results**: The program successfully integrated frailty screening, multidomain interventions, and resident participation through Community Health Centers. Pre- and post-program assessments demonstrated significant improvements in handgrip strength and chair-stand performance, alongside reductions in depression and social isolation (p<0.001). Community Health Groups and Community Health Leaders supported post-program health activities, while HAHA Health Partners facilitated program implementation and ongoing resident activities.

**Conclusions**: The Busan model integrates frailty screening, multidomain interventions, and resident-participatory ongoing management through Community Health Centers. The Haeundae-gu experience demonstrates the feasibility of this approach in community settings, offering a practical foundation to expand community-based frailty prevention and inform future national policies on frailty prevention and community integrated care.

**Key messages**

**① What is known previously?**

Frailty is a multidimensional condition involving physical, psychological, and social factors and can be prevented—or its progression delayed—through multidomain interventions that include exercise, nutrition, cognitive activities, and social engagement. However, practical experience in the Republic of Korea remains limited regarding community-based systems that can sustainably deliver these interventions and support the maintenance of healthy behaviors after program completion.

**② What new information is presented?**

The Busan Health UP Program utilizes Community Health Centers as community-level health management hubs to screen for frailty, provide multidomain interventions primarily for individuals with pre-frailty, and connect participants to long-term management through Community Health Groups.

**③ What are implications?**

Community-based frailty prevention must evolve beyond short-term health promotion programs into a continuous model that integrates frailty screening, multidomain interventions, resident-led self-help activities, participation by trained community members, and reassessment through community-level health management hubs. This operational framework may be adapted to various local contexts by leveraging existing community resources, even in areas without Community Health Centers.

## Introduction

As the Republic of Korea rapidly enters a super-aged society, extending healthy life expectancy and maintaining functional ability in older age are emerging as critical tasks for national healthcare. While the increase in life expectancy reflects advancements in medical technology and improvements in chronic disease management, it simultaneously brings new challenges, such as functional decline and disability in older age, and an increasing demand for long-term care. Therefore, in a super-aged society, prevention-oriented health management strategies are crucial that not only diagnose and treat diseases but also support older adults in maintaining independent daily living for as long as possible.

Frailty refers to a state of increased vulnerability to external stressors due to a decline in physiological reserve and resilience associated with aging and is associated with an increased risk of falls, hospitalization, disability, and long-term care [1,2]. However, frailty is an important target for prevention because it is not simply an inevitable consequence of aging, but is rather a state wherein progression can be delayed or function restored if interventions are made at the appropriate time. In particular, the pre-frailty stage between a healthy state and frailty can be seen as a critical window for intervention before functional decline becomes established.

Frailty cannot be explained solely by a simple decline in muscle strength. Because decreased physical activity, muscle loss, malnutrition, depression, cognitive decline, and social isolation can interact to accelerate functional decline, frailty prevention also requires an approach that manages multiple domains together, rather than targeting only a single risk factor. The World Health Organization presents a function-centered health management approach to maintain the intrinsic capacity of older adults through Integrated Care for Older People, and the FINGER study has also suggested the potential of multidomain interventions, combining exercise, nutrition, cognitive training, and risk factor management [3,4].

However, apart from the evidence on the effectiveness of multidomain interventions, who, where, and how to sustain them in the actual community remains another issue. Although various senior health promotion programs are operated centered on public health centers, they often end as education or exercise programs of a certain duration, and it is not easy to establish an operational structure that supports residents in sustaining the healthy behaviors learned in the programs in their daily lives. In particular, since frailty prevention relies more on repetitive healthy behaviors and social participation than on one-off education, ongoing management plans following short-term programs need to be considered together.

Busan Metropolitan City has operated Community Health Centers as organizations within public health centers so that residents can access health management services within their neighborhoods. The Community Health Center is a community health management hub established and operated to ensure that the public health center’s community health programs can be delivered close to where residents live. It conducts health measurements and counseling, health promotion programs, and resident organization activities for local residents, and is responsible for identifying community health issues and resident needs and linking them to necessary health and welfare resources.

Busan Metropolitan City utilized the operational foundation of these Community Health Centers to promote the Health UP Program for community-based frailty prevention starting in 2024. The Health UP Program established its basic focus on frailty screening, multidomain interventions, and ongoing management through resident activities after the program ended. Haeundae-gu implemented this approach in four Community Health Centers in Banyeo 2-dong, Banyeo 3-dong, Bansong 1-dong, and Bansong 2-dong.

Prior to the introduction of the Health UP Program, Hae­undae-gu already had experience with resident participation and resident organizations formed through the Healthy Bansong Project and the Healthy Community Project. This foundation was utilized to apply Busan Metropolitan City’s project to the local community and link it to resident activities after the program ended.

This article outlines the structure and operational methods of Busan Metropolitan City’s Health UP Program, and presents the implementation experiences at the four Community Health Centers in Haeundae-gu along with the pre- and post-program assessment data collected during the project operation from 2024 to 2025. In addition, we aim to examine the operational direction and policy implications of community-centered frailty prevention projects.

## Methods

### 1. Basic Operational Structure of the Health UP Program

The basic operational process of the Health UP Program consisted of identifying local residents and conducting frailty screening; multidomain interventions in the Health UP Class focusing on the pre-frail group; sustaining the practice of healthy behaviors through resident activities, such as Community Health Groups, after the program ended; and re-evaluation and linkage to local resources as needed. Based on the frailty screening test, the normal group was linked to self-management, existing small health groups, and public health center and Community Health Center programs, while the pre-frail group received more active preventive interventions and ongoing management through participation in the Health UP Class and the formation of Community Health Groups. Finally, for the frail group, linkages to local resources, such as collaborating hospitals, mental health and welfare centers, and home visit health management, were established, and education and counseling for self-management were supported.

### 2. Operating Personnel and Roles of the Community Health Center

The Community Health Center serves as a neighborhood-level health management hub for the public health center’s community health programs and provides health counseling, health assessments, healthy lifestyle programs, and resident participation activities. The basic operating staff consists of a public health official (public official), a community nurse (fixed-term employee), and a community health coordinator (appointed), who are respectively responsible for the administrative operation and coordination of the project, professional health management, and support for resident organization and field activities (Table 1).

The public health official oversees the overall planning and operation of the Community Health Center project, and coordinates program plan formulation, linkage with related programs within the public health center, and cooperation with community organizations. Community nurses are responsible for health counseling and chronic disease management and education for residents, and in the Health UP Program, they perform professional health management, such as frailty-related testing, physical measurements, and health counseling, based on test results. Community health coordinators maintain continuous contact with residents to identify target participants, promote resident participation in health activities, and support the operation of Health UP Classes, as well as the activities of resident organizations and small health groups.

Based on this existing three-person operational system, the Health UP Program did not establish a separate dedicated organization for frailty prevention, but integrated frailty screening, multidomain interventions, and ongoing management functions into the existing functions of the Community Health Center.

### 3. Linkage of Resident Participation Personnel and Resident Organizations

In the Health UP Program, the resident workforce and resident organizations, such as HAHA Health Partners, Com­munity Health Groups, and the Community Health Leaders, were linked with the core operating staff of the Community Health Center. They are not the regular operating staff of the Community Health Center, and each has a different background of formation, mode of participation, and role (Table 2).

A Community Health Group is a self-help group formed by those among the graduates of the Health UP Class who wish to sustain the healthy behaviors acquired in the program. The Community Health Leaders is not an organization newly established for the Health UP Program, but rather a resident health organization formed during the existing community health project, participating in resident health activities, the identification of health-vulnerable residents, and linkage to local resources. HAHA Health Partners are resident operating personnel selected, deployed, and trained through the social service-type senior employment project, supporting frailty screening, the operation of Health UP Classes, the management of individuals subject to reassessment, and small health group activities.

HAHA Health Partners are distinguished from self-help groups or voluntary resident organizations in that they are resident operating personnel with a structured training and activity system. Prior to activities, training was provided on frailty prevention, understanding of the Health UP Program, small health groups and community organizing, and the theory and practice of strength maintenance and strengthening exercises. In addition, they were to support program operations within the scope of their training. Professional health assessment, counseling, and risk determination were handled by professional personnel, such as community nurses, to clearly divide roles.

### 4. Frailty Screening and Health Assessment

The Health UP Program focused on the early detection of frailty risk among community-dwelling older adults and providing preventive interventions, particularly at the pre-frailty stage. The health assessment consisted of domains for frailty, sarcopenia, depression, and social isolation, and was conducted using questionnaire assessments and physical measurements.

For frailty screening, the 5-item questionnaire of the Korean frailty measurement instrument was used, classifying a score of 0 as normal, 1–2 as pre-frailty, and 3–5 as frailty. For sarcopenia screening, the Korean version of the sarcopenia screening questionnaire (SARC-F) was used, and appendicular muscle mass and skeletal muscle mass index (SMI) through body composition analysis, grip strength test, and the time to complete the 5-times chair stand test were evaluated together. For SARC-F, a total score of 4 or higher was classified as suspected sarcopenia. The physical measurement criteria for sarcopenia were defined as an SMI of less than 7.0 kg/m^2^ for men and less than 5.7 kg/m^2^ for women, grip strength of less than 28 kg for men and less than 18 kg for women, and 12 seconds or longer for the 5-times chair stand test.

In the emotional and social domains, a 15-item depression scale and the 3-item UCLA Loneliness Scale were utilized. When selecting participants, not only the test results but also the residents’ willingness to participate and desire for change were considered. The community nurse, community health coordinator, and public health official consulted to determine the final participants. Through this approach, participants for the Health UP Class were selected, focusing on the pre-frail group, and they were linked to self-management, small health groups, or other health and welfare resources depending on their individual health status.

### 5. Multidomain Intervention of the Health UP Class

The Health UP Class is a group-based multidomain intervention program operated by selecting participants belonging to the pre-frail group based on frailty screening results. The number of participants per center was approximately 10–12, and the program was operated once a week for a total of 10 weeks. The program integrated exercise, nutrition, combined cognitive activities, and social activities to manage physical, nutritional, cognitive, and social factors that affect frailty.

The exercise domain was structured around exercises to maintain and strengthen muscle strength, with the goal of maintaining and improving muscle strength and physical function in older adulthood, and was implemented in conjunction with cognitive-motor dual-task exercises that involve performing cognitive tasks and physical activities simultaneously and respiratory muscle exercises. Rather than providing one-time instruction on new exercises, the exercises learned in previous sessions were repeated before new movements were added in a stepwise manner, enabling participants to continue exercising independently even after the program concluded.

In the nutrition domain, education was provided on geriatric nutrition management and the importance of protein intake, while concurrently conducting activities that participants could apply in their daily lives, such as preparing healthy meals and hands-on cooking practice. For the cognitive domain, cognitive-motor dual-task exercises that combine physical activity and cognitive tasks were utilized along with games, and after the participants became familiar with the activities, new tasks were added in a stepwise manner. In the social domain, the program was structured to include community activities, interactions among participants, and outdoor activities, ensuring that program participation itself serves as an opportunity for social interaction and relationship building.

During the program period, the participants were encouraged to self-monitor whether they practiced exercise and nutrition so that the educational content could be repeated in their daily lives. Program operation involved the Community Health Center public health official, community nurse, and community health coordinator, while relevant specialized personnel participated in specialized areas such as nutrition education. HAHA Health Partners supported contacting participants, managing attendance, preparing and facilitating or assisting with the program, and repeated exercise and the practice of healthy behaviors. The detailed structure of the 10-week program is presented in Table 3.

### 6. Ongoing Management after Program Completion

The Health UP Program operated a post-program management process to ensure that participants sustain the healthy behaviors they acquired in the program even after the conclusion of the 10-week Health UP Class. Those who completed the program and wished to do so were encouraged to form Community Health Groups or participate in existing small health groups to continuously practice the exercise and dietary habits learned in the program, while the Community Health Center supported resident-led self-help activities by providing activity spaces and health information.

For residents who do not participate in Community Health Groups or whose use of the center is not sustained, visits and wellness checks by existing resident organizations, such as the Community Health Leaders, and encouragement to visit the center were utilized. In addition, linkages to public health center programs or local resources were provided based on individual health conditions and needs. In cases requiring additional management, such as sarcopenia, functional decline, depression, or social isolation, individuals were guided or linked to health, medical, and welfare resources, including collaborating medical institutions, mental health and welfare centers, administrative welfare centers, and welfare centers.

Frailty screening and the Health UP Class did not end with a one-time evaluation and intervention, but were linked to re-evaluation. Intervention program participants underwent re-evaluation after a certain period of time following their pre- and post-program assessment, and were subsequently scheduled for regular reassessment. Depending on the assessment results, they were linked to small health groups, resident activities, public health center programs, and community resources. Through this approach, short-term program participation was designed to lead to sustained health management within their living area.

## Results

### 1. Application and Operational Progress in Haeundae-gu

Haeundae-gu is an area with large disparities in demographic structure and health status within the region, where some areas of Bansong-dong and Banyeo-dong have a high proportion of the elderly population and exhibit relatively vulnerable characteristics in terms of health behaviors and socioeconomic aspects. In 2024, Haeundae-gu implemented the Busan Metropolitan City Health UP Program at the four Community Health Centers of Banyeo 2-dong, Banyeo 3-dong, Bansong 1-dong, and Bansong 2-dong, which have been operated focusing on these areas. In these areas, even before the introduction of the Health UP Program, resident-participatory health programs had been operated through the Healthy Bansong Project and the Healthy Community Project, and experience in resident health activities and resident organizations, including the Community Health Leaders, had been accumulated. Rather than being the development process of the Health UP Program itself, this existing foundation was utilized as a basis for applying the project established by Busan Metropolitan City to the Haeundae-gu community and linking it to post-program resident activities.

Although the program was initially structured as a 12-week course, it was adjusted to a 10-week course during actual operation, and the 10-week program was subsequently applied as the basic operational unit. In 2025, the Health UP Class was operated twice a year, divided into the first and second terms. Each dong Community Health Center applied the basic framework of frailty screening and multidomain interventions proposed by Busan Metropolitan City but operated while considering local conditions such as the center’s personnel and space, participant characteristics, and existing resident activities. The resident organizations and resident participation experiences established through existing community health programs were utilized to connect Health UP Class participants to resident activities after the program ended.

### 2. Ongoing Management Based on Resident Participation

Applying the ongoing management structure of the Busan Metropolitan City Health UP Program, Haeundae-gu formed Community Health Groups after the completion of the Health UP Class and linked them with the existing Community Health Leaders. As of 2026, a total of 29 Community Health Groups are operating, including 7 in Banyeo 2-dong, 7 in Banyeo 3-dong, 7 in Bansong 1-dong, and 8 in Bansong 2-dong.

The Community Health Leaders is a resident organization formed during the existing community health project, participating in healthy lifestyle practices, identifying and visiting health-vulnerable residents, encouraging the use of the Community Health Center, and linking to local resources. As of 2026, a total of 14 groups are active, including 5 in Banyeo 2-dong, 4 in Banyeo 3-dong, 3 in Bansong 1-dong, and 2 in Bansong 2-dong.

In 2025, in connection with the social service-type senior employment project, a total of eight HAHA Health Partners were deployed, with two placed at each of the four Community Health Centers. After completing the training hosted by Busan Metropolitan City and the capacity-building training of Haeundae-gu, the Health Partners supported contacting Health UP Class participants and managing their attendance, supporting program preparation and progress, supporting repeated exercise activities, managing targets for frailty screening and reassessment, and supporting small health group activities. Professional health assessments and counseling were handled by professional personnel such as community nurses, while HAHA Health Partners supported project operations within the scope of their training.

This resident participation foundation was utilized as an operational framework to sustain healthy lifestyle practices by connecting the self-help activities of Community Health Groups with the support of existing resident organizations after the completion of the Health UP Class.

### 3. Pre- and Post-program Changes in Program Participants

To examine the field operation results of the Health UP Program, we analyzed the pre- and post-program measure­ment data of the participants in the Health UP Class at the Banyeo 2-dong, Banyeo 3-dong, Bansong 1-dong, and Bansong 2-dong Community Health Centers in Haeundae-gu in 2024 and 2025. Of the 150 total registration records, 140 cases with both pre- and post-program data available for key indicators were extracted, and 139 participants were finally analyzed, excluding one duplicate participant. However, for appendicular muscle mass, 137 participants whose pre- and post-measurement values were both confirmed were analyzed. The average age of the subjects was 75.6±5.8 years, with 131 women (94.2%) and 8 men (5.8%).

Comparing the results before and after participating in the program, grip strength increased and the time to complete the 5-times chair stand test was shortened, showing significant improvements in some indicators of physical function. Additionally, depression and social isolation scores also significantly decreased, confirming positive changes in psychosocial indicators as well (p<0.001) (Table 4).

On the other hand, appendicular muscle mass significantly decreased at the post-program assessment. However, because outliers deemed to be data entry errors were identified in some data and the measurement environment and repeated measurement conditions were not fully controlled, the results should be interpreted with caution.

These analytical data were not separately designed and collected for research purposes, but were collected during the operation and monitoring of a community-based program and were used in this article to supplement the presentation of the program’s operational results.

## Discussion

### 1. Significance of Community-based Frailty Prevention Projects

The importance of a multidomain approach for frailty prevention has been suggested in previous studies [3-5]. The significance of the Health UP Program lies not in developing a new exercise method or frailty assessment tool, but rather in connecting frailty screening, multidomain interventions, resident self-help activities, and re-evaluation into a single continuous operational system by utilizing the existing Community Health Centers as living-area hubs.

### 2. Importance of Ongoing Management after Program Completion

In frailty prevention projects, what is more important than the program period itself is how well the behaviors acquired in the program are sustained in subsequent daily life. Even if short-term intensive exercise or nutritional education is provided, if the activities stop upon program completion, it is difficult to achieve long-term functional maintenance. In the Health UP Program, Community Health Groups functioned as a mechanism linking the short-term program with the daily practice of healthy behaviors.

However, data systematically tracking the sustainability, participation frequency, and long-term health outcomes of Community Health Groups are not yet sufficient. Therefore, in the future, it is necessary to evaluate not only changes in physical function at the time of program completion but also indicators such as Community Health Group participation rates, whether physical activity is sustained, re-evaluation results, and entry into long-term care after 6 months or 1 year.

### 3. Resident Participation Structure and the Potential of HAHA Health Partners

In the Haeundae-gu experience, Community Health Groups, the Community Health Leaders, and HAHA Health Partners share the commonality of resident participation, but are classified as self-help groups, existing resident organizations, and trained resident operating personnel, respectively.

As such, the approach of distinguishing between voluntary resident organizations and resident operating personnel with specific duties while utilizing them in a complementary manner is significant in terms of the sustainability of community health programs. While the approach wherein professional personnel, such as nurses, nutritionists, and exercise specialists, directly conduct all activities has advantages in terms of expertise, workforce and cost constraints must be considered to continuously operate large-scale programs in the community. Conversely, it is also inappropriate to entrust professional health management tasks solely to untrained residents.

Therefore, the role of the resident workforce, such as HAHA Health Partners, should be understood not as a replacement for professional personnel, but as an appropriate division of work. Health assessment and counseling, risk determination, and professional intervention are handled by health professionals, while standardized questionnaire support, participant contact, attendance management, support for repeated exercise activities, encouraging the practice of healthy behaviors, and supporting small health groups can be handled by a sufficiently trained resident workforce.

During the operation of HAHA Health Partners, lack of training and lack of individual preparation were presented as difficulties faced by participants. This finding demonstrates that sustainability is not secured merely by deploying resident workforce. It requires selection, basic training, repeated training, field practice, establishing the scope of roles, and supervision by professional personnel. In particular, it is important to clarify the roles among senior employment project operating agencies, public health centers, and Community Health Centers and to assign participants considering their health status and activity capacity.

### 4. The Significance of the Experiences of Busan Metropolitan City and Haeundae-gu

Busan Metropolitan City established the basic direction of the Health UP Program based on the Community Health Centers, and Haeundae-gu utilized existing community health projects and resident organizations to apply it at four Community Health Centers. This experience shows that even if metropolitan governments present a standardized project framework, actual operation and ongoing management can be adjusted based on the local resources and resident participation of basic local governments.

### 5. Applicability to Other Regions

Applying the Health UP Program to other regions does not require establishing new facilities identical to Busan’s Community Health Centers. While Community Health Centers serve as neighborhood-level health management hubs in the Health UP Program, similar functions could be performed in other regions by public health centers, health subcenters, community health promotion centers, primary health care posts, or other existing public facilities that are readily accessible to local residents.

When applying the program to other regions, the key is not to replicate the name or facilities of Community Health Centers, but to ensure the core functions of a neighborhood-level hub, standardized frailty screening, multidomain interventions, and post-program resident activities and reassessment. In addition, it is necessary to distinguish the roles of professional personnel and trained resident workforce and establish an operational system that links them with existing local health, medical, and welfare resources.

Regions with well-developed existing resident organizations can utilize them, as in Haeundae-gu District. Regions without such organizations can gradually form small self-help groups centered on graduates of the frailty prevention and management class. In regions without the conditions to link with the senior employment project, health leaders from public health centers, volunteers, or local community activists can be utilized. The key is not to replicate Busan’s organizational names and operating methods, but to restructure the available local workforce and space to align with frailty prevention functions.

This perspective can also be linked to community integrated care policies. Frailty is not an issue that should be managed only after medical diseases occur. Rather, it needs to be detected and managed in the community before progressing to disability and long-term care. If the frailty prevention project develops as a link between public health programs and medical, welfare, and care services in the future, more continuous support will be possible in accordance with functional changes progressing from healthy older adults to pre-frailty, frailty, and care-requiring stages.

### 6. Limitations of Project Evaluation and Future Tasks

The pre- and post-program results of the participants presented in this article were based on data accumulated during the implementation of community programs and should be interpreted with caution. Because this is a single-group analysis without a control group, the differences before and after participating in the program cannot be definitively concluded to be the causal effects of the Health UP Program. Furthermore, because most participants were women, there is a limitation in generalizing the results to all community-dwelling older adults.

In addition, because the measurement conditions and input data for the body composition data collected in the community field were not sufficiently standardized, caution is required when interpreting the results. In the future, it is necessary to strengthen data entry verification and the standardization of measurement conditions.

The performance indicators of the project also need to be expanded. If we track not only current short-term indicators, such as grip strength, the chair stand test, depression, and social isolation, but also long-term changes in physical activity, falls, healthcare utilization, entry into long-term care, and activities of daily living, the policy effects of community-based frailty prevention programs can be evaluated more objectively.

Moreover, for the sustainability of the Health UP Program, it is necessary not only to expand the number of participants but also to standardize the competencies and roles of the personnel implementing the project. It is important to specify the scope of work that can be handled by community nurses, community health coordinators, nutritionists, exercise specialists, and HAHA Health Partners, and to establish training and supervision systems so that the resident workforce does not handle areas requiring professional judgment.

The Busan Health UP Program is a community-based frailty prevention program that utilizes Community Health Centers as neighborhood-level health management infrastructure within the public health center system to identify frailty risk early among community-dwelling residents, provide multidomain interventions primarily for individuals with pre-frailty, and link participants to Community Health Groups and resident-led activities to support the continuation of healthy behaviors after program completion.

The operational experience in Haeundae-gu demonstrated that this model is applicable in actual communities and can be continuously linked to Community Health Groups and resident participation activities after the program ends. In particular, the Community Health Groups and the Community Health Leaders promoted residents’ voluntary health activities, and HAHA Health Partners supplemented the practical tasks of frailty screening, program operation, and follow-up management. This finding demonstrated the potential for older adults to participate not merely as beneficiaries of health services but as a resident workforce who support the practice of healthy behaviors among their peers.

The key lesson from the Health UP Program and Haeun­dae-gu’s operational experience is the importance of establishing a structure that can sustain frailty prevention efforts in the community, rather than replicating a specific program or facility model. At the core of this structure are neighborhood-level health management hubs that identify and screen residents for frailty and provide multidomain interventions, link program completers to self-help groups and resident organizations, and enable health professionals and trained community members to share roles in supporting continued health activities and reassessment.

Therefore, even in other regions that do not have organizations such as the Community Health Centers in Busan, it is thought that a similar frailty prevention program could be applied if these core functions are configured to suit local conditions by utilizing existing local resources, such as public health centers, community health promotion centers, and primary health care posts. The Busan Metropolitan City Health UP Program could be utilized as a reference model for the future development of national frailty prevention policies and community-integrated care policies.

## Figures and Tables

**Table 1:** Community health center personnel and their roles in the Health UP Program

Category	Basic roles in the Community Health Center	Roles in the Health UP Program
Public health official	Program planning and operation; coordination with the public health center and local organizations; support for resident communities	Overall program management and operational coordination; linkage of participating personnel to local resources
Community nurse	Health counseling; chronic disease management and education; management of residents at health risk	Frailty-related assessments and physical measurements; counseling on results and health management
Community health coordinator	Resident outreach and organization; resident education; support for health leaders and small health groups	Participant outreach; operation of Health UP Classes; support for resident activities, including Community Health Groups

**Table 2:** Resident personnel and community organizations linked to the Health UP Program

Category	HAHA Health Partner	Community Health Group	Community Health Leaders
Type	Trained resident program personnel	Self-help group mainly comprising Health UP graduates	Existing resident health organization
Participation	Linked to the senior employment program; training and placement	Formed mainly by interested program graduates	Organized through existing community health programs
Main roles	Support for frailty assessments, program operation, reassessment, and small-group activities	Continuation of healthy lifestyle practices, including exercise and nutrition	Resident health activities; identification and support of health-vulnerable residents; linkage to local resources
Relationship to Health UP	Support for program operation and ongoing management	Ongoing management after program completion	Community linkage via the existing resident-based network

**Table 3:** Structure of the 10-week Health UP Class Program

Session	Domain	Topic	Content	Personnel
1	Program orientation and introduction	Opening session	Icebreaker: self-introduction and reasons for participation Program orientation and introduction What is frailty?	Community health coordinator, public health official, community nurse, HAHA Health Partner
	Exercise	Learning muscle-strengthening exercises (beginner level)	Basic movements - Exercises that can be performed independently - Learn and practice each movement step by step Precautions during exercise Instructions on using educational materials and the practice checklist	Community health coordinator, HAHA Health Partner
2	Nutrition	Diet for maintaining muscle strength	Review basic movements together Eat right with proper knowledge (introduction to protein drinks) Instructions on using the practice checklist	Public health center dietitian, HAHA Health Partner
3	Cognition+ exercise	Keeping the brain young	Practice basic movements together (repetition) Combined cognitive games and exercises	Community health coordinator, HAHA Health Partner
4	Exercise	Learning muscle-strengthening exercises (intermediate level)	Additional movements - Exercises performed with others - Learn and practice each movement step by step Precautions during exercise	Community health coordinator, HAHA Health Partner
5	Community	Living as a member of the community	Review additional movements together Community education (40 minutes)	Community health coordinator, HAHA Health Partner
6	Social activity	Neighborhood outing (outdoor activity)	Practice basic and additional movements together (repetition) Explore the neighborhood - Identify places suitable for exercise - Develop a personal walking route - Learn appropriate walking exercise intensity	Community health coordinator, community nurse, HAHA Health Partner
7	Nutrition	Diet for maintaining muscle strength	Practice basic and additional movements together (repetition) Review adherence to healthy eating habits Prepare healthy food together	Community health coordinator, community nurse, HAHA Health Partner
8	Cognition+ exercise	Keeping the brain young	Practice basic and additional movements together (repetition) Combined cognitive games and exercises	Community health coordinator, HAHA Health Partner
9	Exercise	Learning exercises to strengthen respiratory muscles	Practice basic and additional movements together (repetition) Learn and practice respiratory muscle-strengthening exercises step by step - Using sound level meters, straws, singing exercises, etc.	Community health coordinator, HAHA Health Partner
10	Social activity	Workshop for small-group activities	Practice basic and additional movements together (repetition) Review respiratory muscle-strengthening exercises together Discuss plans for future small health-group activities	Community health coordinator, HAHA Health Partner
	Closing	Graduation ceremony	Remarks by the public health center director and presentation of certificates Presentation of achievements and awards	Public health official, public health center staff

**Table 4:** Changes in pre- and post-intervention indicators among participants in the Health UP Program

Indicator	n	Pre-intervention mean	Post-intervention mean	Mean change	p-value
Appendicular skeletal muscle mass (kg)	137	18.45±4.41	17.47±2.34	–0.97±3.84	0.028
Handgrip strength (kg)	139	20.72±5.23	21.62±5.31	0.90±3.37	<0.001
Chair stand test (sec)	139	10.32±3.50	9.07±3.28	–1.25±3.17	<0.001
Depression (score)	139	4.04±3.59	2.83±3.31	–1.20±2.79	<0.001
Social isolation (score)	139	4.25±1.58	3.88±1.41	–0.37±1.26	<0.001

Changes were calculated as post-intervention minus pre-intervention values. Data are presented as mean±standard deviation. For the chair stand test, depression, and social isolation, lower scores or shorter times indicate improvement. p-values were obtained using the two-sided Wilcoxon signed-rank test.
