# Supplementary material for: Need for and Key Outcomes of a Community-based Frailty Prevention Program: The Busan Health UP Program and Operational Experience in Haeundae-gu
Source: Public Health Wkly Rep. 2026 Oct 29;19(41 Suppl):59–82. doi: 10.56786/PHWR.2026.19.41suppl.4 (PMC13724137; doi:10.56786/PHWR.2026.19.41suppl.4)
Supplement: Translation

# Translation: 지역사회 중심 노쇠예방사업의 필요성과 주요 성과: 부산광역시 건강UP 사업과 해운대구 운영 경험

**Authors:** 여현 안

**Affiliations:** 부산광역시 해운대구 보건소

**Keywords:** 노쇠, 건강UP 사업, 마을건강센터, 지역사회 주민 참여

## Abstract

**목적:**

부산광역시가 마을건강센터를 기반으로 2024년부터 추진한 건강UP 사업의 구성과 운영방법을 정리하고, 해운대구 4개 마을건강센터에서의 적용과 운영 경험 및 사업 결과를 바탕으로 지역사회 중심 노쇠예방사업의 발전 방향과 정책적 시사점을 제시하고자 하였다.

**방법:**

2024년부터 추진된 건강UP 사업의 노쇠 조기선별, 운동‧영양‧인지‧사회참여를 포함한 다영역 중재와 건강동아리, 건강지킴이단 및 하하건강파트너를 연계한 주민 참여형 지속관리 구조를 고찰하였다. 또한 해운대구 건강UP 사업 참여자 139명의 사전‧사후 평가자료를 양측 Wilcoxon 부호순위검정으로 분석하였다.

**결과:**

건강UP 사업은 마을건강센터를 중심으로 노쇠 조기선별, 다영역 중재 및 주민 참여형 지속관리를 연계한 지역사회 노쇠예방 전달체계를 구축하였다. 참여자의 사전‧사후 평가 결과, 악력과 의자 일어서기 수행능력이 유의하게 개선되었으며, 우울과 사회적 고립감도 유의하게 감소하였다(p<0.001). 또한 건강동아리와 건강지킴이단을 통해 프로그램 종료 이후에도 주민의 건강활동 지속을 지원하고, 하하건강파트너와 연계하여 주민 참여형 지속관리체계를 확장하였다.

**결론:**

부산형 지역사회 노쇠예방모델은 마을건강센터를 중심으로 노쇠 조기선별, 다영역 중재 및 주민 참여형 지속관리체계를 통합한 지역사회 실천모델이다. 해운대구의 운영 경험과 사업 결과는 이러한 모델의 현장 적용 가능성을 보여주었으며, 향후 지역사회 노쇠예방사업의 확산과 국가 노쇠예방정책 및 지역사회 통합돌봄 정책을 위한 지역사회 기반 모델로 활용될 수 있을 것으로 기대된다.

**핵심요약**

**① 이전에 알려진 내용은?**

노쇠는 신체적‧정신적‧사회적 요인이 복합적으로 작용하는 상태로, 운동‧영양‧인지 및 사회적 영역을 포함한 다영역 중재를 통해 예방하거나 진행을 지연할 수 있다. 그러나 이러한 중재를 지역사회에서 지속적으로 제공하고, 프로그램 종료 이후에도 건강행동을 유지하도록 지원하는 운영체계에 대한 국내 경험은 아직 충분하지 않다.

**② 새로이 알게 된 내용은?**

부산광역시 건강UP 사업은 소생활권 건강관리 거점인 마을건강센터를 활용하여 노쇠선별을 하고 전노쇠군 중심의 다영역 중재를 제공하고 건강동아리를 통한 지속관리를 연계하였다.

**③ 시사점은?**

지역사회 노쇠예방은 단기간의 건강증진 프로그램을 넘어 생활권 건강관리 거점을 중심으로 노쇠선별, 다영역 중재, 주민 자조활동과 주민 인력의 참여 및 재평가를 연속적으로 구성하는 방향으로 발전할 필요가 있다. 이러한 운영구조는 마을건강센터가 없는 지역에서도 기존 자원을 활용하여 지역 여건에 맞게 적용할 수 있는 실천 모델로 활용될 수 있다.

## 서 론

우리나라는 빠른 속도로 초고령사회에 진입하면서 건강수명의 연장과 노년기 기능 유지가 국가 보건의료의 중요한 과제로 대두되고 있다. 평균수명의 증가는 의료기술의 발전과 만성질환 관리 수준의 향상을 반영하지만, 동시에 노년기의 기능 저하와 장애, 장기요양 수요 증가라는 새로운 과제를 동반한다. 따라서 초고령사회에서는 질병의 진단과 치료뿐만 아니라 노인이 가능한 오랫동안 독립적인 일상생활을 유지하도록 지원하는 예방 중심의 건강관리 전략이 중요하다.

노쇠(frailty)는 노화에 따라 생리적 예비능과 회복력이 감소하여 외부 스트레스에 대한 취약성이 증가한 상태를 의미하며 낙상, 입원, 장애 및 장기요양 위험의 증가와 관련된다[1,2]. 그러나 노쇠는 노화에 따른 불가피한 결과만을 의미하는 것이 아니라 적절한 시기에 개입하면 진행을 늦추거나 기능을 회복할 가능성이 있는 상태라는 점에서 중요한 예방 대상이다. 특히 건강한 상태와 노쇠 사이의 전노쇠(pre-frailty) 단계는 기능 저하가 고착되기 전에 개입할 수 있는 중요한 시기로 볼 수 있다.

노쇠는 단순한 근력 저하만으로 설명되지 않는다. 신체활동 감소와 근감소, 영양불량, 우울, 인지기능 저하 및 사회적 고립 등이 서로 영향을 미치면서 기능 저하를 촉진할 수 있기 때문에 노쇠예방 역시 하나의 위험요인만을 대상으로 하기보다 여러 영역을 함께 관리하는 접근이 필요하다. 세계보건기구(World Health Organization)는 노인 통합관리지침(Integrated Care for Older People)을 통해 노인의 내재적 역량(intrinsic capacity)을 유지하는 기능 중심의 건강관리 접근을 제시하고 있으며, FINGER 연구에서도 운동, 영양, 인지훈련과 위험요인 관리 등을 결합한 다영역 중재의 가능성이 제시되어 왔다[3,4].

그러나 다영역 중재의 효과에 대한 근거와 별개로 이를 실제 지역사회에서 누가, 어디에서, 어떤 방식으로 지속할 것인가는 또 다른 문제이다. 보건소를 중심으로 다양한 노인 건강증진 프로그램이 운영되고 있으나 일정 기간의 교육이나 운동 프로그램으로 종료되는 경우가 많으며, 프로그램에서 습득한 건강행동을 주민이 생활 속에서 지속하도록 지원하는 운영구조를 마련하기는 쉽지 않다. 특히 노쇠예방은 일회성 교육보다 반복적인 건강행동과 사회참여가 중요하므로 단기 프로그램 이후의 지속관리 방안이 함께 고려될 필요가 있다.

부산광역시는 주민이 거주하는 소생활권에서 건강관리 서비스를 이용할 수 있도록 보건소 내 조직으로 마을건강센터를 운영해 왔다. 마을건강센터는 보건소의 지역보건사업이 주민의 생활권 가까이에서 이루어질 수 있도록 설치‧운영되는 지역사회 건강관리 거점이다. 지역주민을 대상으로 건강측정과 상담, 건강증진 프로그램, 주민 조직 활동 등을 수행하며, 지역의 건강문제와 주민의 요구를 파악하고 필요한 보건‧복지자원과 연계하는 기능을 담당한다.

부산광역시는 이러한 마을건강센터의 운영 기반을 활용하여 2024년부터 지역사회 노쇠예방을 위한 건강UP 사업을 추진하였다. 건강UP 사업은 노쇠선별, 다영역 중재와 프로그램 종료 후 주민활동을 통한 지속관리를 기본 방향으로 하였으며, 해운대구는 반여2동, 반여3동, 반송1동 및 반송2동의 4개 마을건강센터에서 이를 시행하였다.

해운대구에는 건강UP 사업 도입 이전부터 건강반송사업과 건강마을사업 등을 통해 형성된 주민 조직과 주민 참여 경험이 있었다. 이러한 기반은 부산광역시의 사업을 지역사회에 적용하고 프로그램 종료 이후 주민활동으로 연결하는 데 활용되었다.

본 글에서는 부산광역시 건강UP 사업의 구성과 운영방법을 정리하고, 해운대구 4개 마을건강센터에서의 적용 경험과 2024–2025년 사업 운영 과정에서 수집된 사전‧사후 평가자료를 제시하였다. 이를 바탕으로 지역사회 중심 노쇠예방사업의 운영 방향과 정책적 시사점을 고찰하였다.

## 방 법

### 1. 건강UP 사업의 기본 운영구조

건강UP 사업의 기본 운영과정은 지역주민의 발굴과 노쇠선별, 전노쇠군을 중심으로 한 건강UP 교실의 다영역 중재, 프로그램 종료 후 건강동아리 등 주민활동을 통한 건강생활실천의 지속, 재평가 및 필요에 따른 지역자원 연계로 구성되었다. 노쇠선별검사상 정상군은 자가관리와 기존 건강소모임 및 보건소‧마을건강센터 프로그램으로 연계하고, 전노쇠군은 건강UP 교실 참여와 건강동아리 형성 등을 통해 보다 적극적인 예방적 중재와 지속관리가 이루어지도록 하였다. 마지막으로 노쇠군에게는 협력병원, 정신건강복지센터, 방문건강관리 등 지역자원을 연계하고 자가관리를 위한 교육 및 상담을 지원했다.

### 2. 마을건강센터의 운영인력과 역할

마을건강센터는 보건소 지역보건사업의 소생활권 건강관리 거점으로, 주민의 건강상담과 건강측정, 건강생활실천 프로그램 및 주민 참여 활동 등을 수행한다. 기본 운영인력은 담당자(공무원), 마을간호사(기간제) 및 마을건강활동가(위촉직)로 구성되며, 이들은 각각 사업의 행정적 운영과 조정, 전문적인 건강관리, 주민 조직화와 현장 활동 지원의 역할을 담당한다(표 1).

담당자는 마을건강센터 사업의 기획과 운영을 총괄하고 사업계획 수립, 보건소 내 관련 사업과의 연계 및 지역사회 기관과의 협력을 조정한다. 마을간호사는 주민의 건강상담과 만성질환 관리‧교육을 담당하며, 건강UP 사업에서는 노쇠 관련 검사와 신체측정, 검사결과에 따른 건강상담 등 전문적인 건강관리를 수행한다. 마을건강활동가는 주민과 지속적으로 접촉하면서 대상자를 발굴하고 주민의 건강활동 참여를 촉진하며, 건강UP 교실 운영과 주민 조직 및 건강소모임 활동을 지원한다.

건강UP 사업은 이러한 기존 3인 운영체계를 기반으로 별도의 노쇠예방 전담조직을 설치하지 않고 마을건강센터의 기존 기능에 노쇠선별, 다영역 중재 및 지속관리 기능을 결합하였다.

### 3. 주민 참여 인력 및 주민 조직의 연계

건강UP 사업에서는 마을건강센터의 기본 운영인력과 함께 하하건강파트너, 건강동아리 및 건강지킴이단 등 주민 인력과 주민 조직을 연계하였다. 이들은 마을건강센터의 상시 운영인력이 아니며 각각의 형성 배경과 참여방식 및 역할이 다르다(표 2).

건강동아리는 건강UP 교실 수료자 가운데 희망자가 프로그램에서 습득한 건강행동을 지속하기 위해 구성하는 자조모임이다. 건강지킴이단은 건강UP 사업을 위해 새롭게 구성한 조직이 아니라 기존 마을건강사업 과정에서 형성된 주민 건강조직으로, 주민 건강활동과 건강취약주민 발굴 및 지역자원 연계 등에 참여한다. 하하건강파트너는 사회서비스형 노인일자리사업을 통해 선발‧배치하고 교육하는 주민 운영인력으로, 노쇠검사와 건강UP 교실 운영, 재검 대상자 관리 및 건강소모임 활동 등을 지원한다.

하하건강파트너는 일정한 교육과 활동체계를 갖는 주민 운영인력이라는 점에서 자조모임이나 자발적 주민 조직과 구별된다. 활동 전 노쇠예방과 건강UP 사업의 이해, 건강소모임과 주민 조직화, 근력 유지‧강화운동의 이론과 실습 등에 대한 교육을 시행하고, 교육받은 범위에서 사업 운영을 지원하도록 하였다. 전문적인 건강평가와 상담 및 위험판단은 마을간호사 등 전문인력이 담당하여 역할을 구분하였다.

### 4. 노쇠선별 및 건강평가

건강UP 사업은 지역사회 거주 노인의 노쇠 위험을 조기에 발견하고 특히 전노쇠 단계에서 예방적 중재를 제공하는 데 중점을 두었다. 건강평가는 노쇠, 근감소증, 우울 및 사회적 고립 영역으로 구성하고 설문평가와 신체측정을 함께 시행하였다.

노쇠검사는 한국형 노쇠 측정도구 5문항 설문을 이용하여 0점을 정상, 1–2점을 전노쇠, 3–5점을 노쇠로 분류하였다. 근감소증 선별에는 한국형 근감소증 선별 질문지(SARC-F)를 사용하고, 체성분 측정을 통한 사지근육량 및 골격근량지수(skeletal muscle mass index, SMI), 악력, 5회 의자 앉았다 일어서기 수행시간을 함께 평가하였다. SARC-F는 총점 4점 이상을 근감소증 의심으로 평가하였고, 신체측정 기준은 SMI 남성 7.0 kg/m^2^ 미만‧여성 5.7 kg/m^2^ 미만, 악력 남성 28 kg 미만‧여성 18 kg 미만, 5회 의자 일어서기 12초 이상으로 하였다.

정서‧사회적 영역에서는 15문항 우울검사와 3문항 UCLA 외로움 척도를 활용하였다. 대상자 선정 시 검사결과뿐 아니라 주민의 참여 의지와 변화 의지를 함께 고려하였으며, 마을간호사, 마을건강활동가 및 담당자가 협의하여 최종 참여자를 결정하였다. 이를 통해 전노쇠군을 중심으로 건강UP 교실 참여자를 선정하고, 개인의 건강상태에 따라 자가관리, 건강소모임 또는 다른 보건‧복지자원으로 연계하였다.

### 5. 건강UP 교실의 다영역 중재

건강UP 교실은 노쇠선별 결과를 바탕으로 전노쇠군을 중심으로 참여자를 선정하여 운영하는 집단형 다영역 중재 프로그램이다. 센터별 참여인원은 약 10–12명으로 구성하고, 주 1회씩 총 10주간 운영하였다. 프로그램은 노쇠에 영향을 미치는 신체적‧영양적‧인지적‧사회적 요인을 함께 관리할 수 있도록 운동, 영양, 인지복합활동 및 사회적 활동을 통합하여 구성하였다.

운동 영역은 노년기 근력과 신체기능의 유지‧향상을 목적으로 근력 유지‧강화운동을 중심으로 구성하고 인지과제와 신체활동을 동시에 수행하는 인지복합운동과 호흡근운동 등을 함께 실시하였다. 새로운 운동을 일회성으로 교육하기보다 이전 회차에서 학습한 운동을 반복한 뒤 새로운 동작을 단계적으로 추가하여 참여자가 프로그램 종료 이후에도 스스로 운동을 지속할 수 있도록 하였다.

영양 영역에서는 노년기 영양관리와 단백질 섭취의 중요성을 교육하고, 건강식 만들기와 조리실습 등 참여자가 일상생활에서 적용할 수 있는 활동을 병행하였다. 인지 영역은 신체활동과 인지과제를 결합한 인지복합운동과 게임을 활용하고, 참여자가 활동에 익숙해진 후 새로운 과제를 단계적으로 추가하였다. 사회적 영역에서는 공동체 활동과 참여자 간 상호작용, 야외활동 등을 포함하여 프로그램 참여 자체가 사회적 교류와 관계 형성의 기회가 되도록 구성하였다.

프로그램 기간 중에는 운동과 영양 실천 여부를 스스로 점검하도록 하여 교육내용이 일상생활에서 반복되도록 하였다. 프로그램 운영에는 마을건강센터 담당자, 마을간호사 및 마을건강활동가가 참여하고, 영양교육 등 전문영역에는 관련 전문인력이 참여하였다. 하하건강파트너는 참여자 연락과 출석관리, 프로그램 준비와 진행 또는 보조진행, 반복적인 운동 및 건강생활실천을 지원하였다. 구체적인 10주 프로그램 구성은 표 3에 제시하였다.

### 6. 프로그램 종료 후 지속관리

건강UP 사업은 10주간의 건강UP 교실 종료 이후에도 참여자가 프로그램에서 습득한 건강행동을 지속하도록 사후관리 과정을 운영하였다. 수료자 중 희망자는 건강동아리를 구성하거나 기존 건강소모임에 참여하여 프로그램에서 배운 운동과 식생활을 지속적으로 실천하도록 하였으며, 마을건강센터는 활동공간과 건강정보 제공 등을 통해 주민 주도의 자조활동을 지원하였다.

건강동아리에 참여하지 않거나 센터 이용이 지속되지 않는 주민에 대해서는 건강지킴이단 등 기존 주민 조직의 방문과 안부 확인, 센터 방문 독려 등을 활용하고, 개인의 건강상태와 요구에 따라 보건소 프로그램이나 지역자원을 연계하도록 하였다. 근감소증이나 기능 저하, 우울 또는 사회적 고립 등 추가적인 관리가 필요한 경우에는 협력 의료기관, 정신건강복지센터, 행정복지센터, 복지관 등 보건‧의료‧복지자원으로 안내하거나 연계하였다.

노쇠선별과 건강UP 교실은 일회성 평가와 중재로 종료하지 않고 재평가와 연결하였다. 중재프로그램 참여자는 사전‧사후평가 후 일정 기간이 지나 재평가를 시행하고 이후 정기적으로 재검사하도록 하였으며, 평가결과에 따라 건강소모임, 주민활동, 보건소 사업 및 지역사회 자원으로 연계하였다. 이를 통해 단기간의 프로그램 참여가 생활권 내 지속적인 건강관리로 이어지도록 하였다.

## 결 과

### 1. 해운대구 적용과 운영 경과

해운대구는 지역 내 인구구조와 건강수준의 격차가 큰 지역으로, 반송동과 반여동 일부 지역은 고령인구 비율이 높고 건강행태와 사회경제적 측면에서 상대적으로 취약한 특성을 보인다. 해운대구는 이러한 특성을 반영하여 2024년에는 해당 지역을 중심으로 반여2동, 반여3동, 반송1동 및 반송2동의 4개 마을건강센터에서 부산광역시 건강UP 사업을 시행하였다. 이들 지역에는 건강UP 사업 도입 이전부터 건강반송사업과 건강마을사업 등을 통해 주민 참여형 건강사업이 운영되어 왔으며, 건강지킴이단을 비롯한 주민 조직과 주민 건강활동의 경험이 축적되어 있었다. 이러한 기존 기반은 건강UP 사업의 개발과정 자체라기보다 부산광역시가 마련한 사업을 해운대구 지역사회에 적용하고 프로그램 이후 주민활동으로 연계하는 기반으로 활용되었다.

프로그램은 당초 12주 과정으로 구성되었으나 실제 운영과정에서 10주 과정으로 조정되었으며, 이후 10주 프로그램을 기본적인 운영단위로 적용하였다. 2025년에는 1기와 2기로 나누어 연간 2회 건강UP 교실을 운영하였다. 각 동 마을건강센터에서는 부산광역시가 제시한 노쇠선별과 다영역 중재의 기본구조를 적용하되 센터의 인력과 공간, 참여자의 특성 및 기존 주민활동 등 지역 여건을 고려하여 운영하였으며 기존 마을건강사업을 통해 형성되어 있던 주민 조직과 주민 참여 경험은 건강UP 교실 참여자를 프로그램 종료 이후의 주민활동으로 연결하는 데 활용되었다.

### 2. 주민 참여 기반의 지속관리 운영

해운대구에서는 부산광역시 건강UP 사업의 지속관리 구조를 적용하면서 건강UP 교실 종료 후 건강동아리를 구성하고 기존 건강지킴이단과 연계하였다. 2026년 현재 반여2동 7개, 반여3동 7개, 반송1동 7개, 반송2동 8개 등 총 29개의 건강동아리가 운영되고 있다.

건강지킴이단은 기존 마을건강사업 과정에서 형성된 주민 조직으로, 건강생활실천, 건강취약주민 발굴‧방문, 마을건강센터 이용 독려와 지역자원 연계 등에 참여하였다. 2026년 현재 반여2동 5개, 반여3동 4개, 반송1동 3개, 반송2동 2개 등 총 14개가 활동하고 있다.

2025년에는 사회서비스형 노인일자리사업과 연계하여 4개 마을건강센터에 하하건강파트너를 각 2명씩 총 8명 배치하였다. 이들은 부산광역시 주관 교육과 해운대구 역량강화교육 수료 후 건강UP 교실 참여자 연락과 출석관리, 프로그램 준비와 진행 지원, 반복적인 운동활동 지원, 노쇠검사 및 재검 대상자 관리와 건강소모임 활동 등을 지원하였다. 전문적인 건강평가와 상담은 마을간호사 등 전문인력이 담당하고, 하하건강파트너는 교육받은 범위에서 사업 운영을 지원하였다.

이러한 주민 참여 기반은 건강UP 교실 종료 후 건강동아리의 자조활동과 기존 주민 조직의 지원을 연결하여 건강생활실천을 지속하기 위한 운영 기반으로 활용되었다.

### 3. 사업 참여자의 프로그램 사전‧사후 변화

건강UP 사업의 현장 운영 결과를 살펴보기 위하여 2024년과 2025년 해운대구 반여2동, 반여3동, 반송1동 및 반송2동 마을건강센터 건강UP 교실 참여자의 프로그램 참여 전‧후 측정자료를 분석하였다. 전체 등록자료 150건 중 핵심 지표의 사전‧사후 자료가 모두 확인되는 140건을 추출하고, 이 중 중복 참여자 1건을 제외한 139명을 최종 분석하였다. 다만, 사지근육량은 사전‧사후 측정값이 모두 확인되는 137명을 분석하였다. 대상자의 평균 연령은 75.6±5.8세였으며 여성은 131명(94.2%), 남성은 8명(5.8%)이었다.

프로그램 참여 전‧후를 비교한 결과, 악력은 증가하고 5회 의자 일어서기 수행시간은 단축되어 신체기능의 일부 지표에서 유의한 개선이 관찰되었다. 또한 우울과 사회적 고립감 점수도 유의하게 감소하여 정신‧사회적 지표에서도 긍정적인 변화가 확인되었다(p<0.001) (표 4).

반면 사지근육량은 사후평가에서 통계적으로 유의하게 감소하였는데 이는 일부 자료에서 입력 오류로 판단되는 이상치가 확인되었고 측정환경과 반복측정 조건을 완전히 통제하지 못하였으므로, 결과를 신중하게 해석할 필요가 있다.

본 분석자료는 연구목적으로 별도 설계‧수집된 자료가 아니라 지역사회 사업 운영 및 모니터링 과정에서 수집된 자료이며, 본 글에서는 사업 운영 결과를 보완적으로 제시하기 위해 활용하였다.

## 논 의

### 1. 지역사회 노쇠예방사업의 의미

노쇠예방을 위한 다영역 접근의 중요성은 기존 연구들에서 제시되어 왔다[3-5]. 건강UP 사업의 의의는 새로운 운동법이나 노쇠평가도구의 개발에 있다기보다는, 마을건강센터라는 기존 생활권 거점을 활용하여 노쇠선별, 다영역 중재, 주민 자조활동 및 재평가를 하나의 연속적인 운영체계로 연결했다는 데 있다.

### 2. 프로그램 종료 이후 지속관리의 중요성

노쇠예방사업에서 프로그램 기간 자체보다 중요한 것은 프로그램에서 습득한 행동이 이후의 생활 속에서 얼마나 지속되는가이다. 단기간의 집중적인 운동이나 영양교육을 제공하더라도 프로그램 종료와 함께 활동이 중단된다면 장기적인 기능 유지로 이어지기 어렵다. 건강UP 사업에서 건강동아리는 단기 프로그램과 일상적인 건강생활실천을 연결하는 장치로 기능하였다.

다만 건강동아리의 지속 여부와 참여빈도, 장기적인 건강결과를 체계적으로 추적한 자료는 아직 충분하지 않다. 따라서 향후에는 프로그램 종료 시점의 신체기능 변화뿐 아니라 6개월 또는 1년 후 건강동아리 참여율, 신체활동 지속 여부, 재평가 결과 및 장기요양 진입 등의 지표를 함께 평가할 필요가 있다.

### 3. 주민 참여 구조와 하하건강파트너의 가능성

해운대구 경험에서 건강동아리, 건강지킴이단 및 하하건강파트너는 모두 주민 참여라는 공통점이 있으나 각각 자조모임, 기존 주민 조직, 교육받은 주민 운영인력으로 구분된다.

이처럼 자발적 주민 조직과 일정한 직무를 가진 주민 운영인력을 구분하면서 상호보완적으로 활용하는 방식은 지역사회 건강사업의 지속가능성 측면에서 의미가 있다. 간호사, 영양사, 운동전문가 등 전문인력이 모든 활동을 직접 수행하는 방식은 전문성 측면에서 장점이 있으나, 지역사회에서 대규모 사업을 지속적으로 운영하려면 인력과 비용의 제약을 고려해야 한다. 반대로 교육받지 않은 주민에게만 전문적 건강관리 업무를 맡기는 것도 적절하지 않다.

따라서 하하건강파트너와 같은 주민 인력의 역할은 전문인력의 대체가 아니라 업무의 적절한 분담으로 이해해야 한다. 건강평가와 상담, 위험판단 및 전문적 중재는 보건전문인력이 담당하고, 표준화된 설문 지원, 참여자 연락, 출석관리, 반복적인 운동활동 지원, 건강생활실천 독려 및 건강소모임 지원 등은 충분한 교육을 받은 주민 인력이 담당할 수 있다.

하하건강파트너 운영 과정에서 참여자의 애로사항으로 교육 부족과 개인별 준비 부족이 제시되었는데, 이는 주민 인력을 배치하는 것만으로 지속가능성이 확보되는 것이 아니라 선발, 기본교육, 반복교육, 현장 실습, 역할범위 설정 및 전문인력의 감독이 함께 필요하다는 것을 보여준다. 특히 노인일자리사업 수행기관과 보건소, 마을건강센터 사이의 역할을 명확히 하고 참여자의 건강상태와 활동역량을 고려하여 배치하는 것이 중요하다.

### 4. 부산광역시와 해운대구 경험의 의미

부산광역시는 마을건강센터를 기반으로 건강UP 사업의 기본방향을 마련하고, 해운대구는 기존 마을건강사업과 주민 조직을 활용하여 이를 4개 마을건강센터에서 적용하였다. 이 경험은 광역단체가 표준적인 사업 틀을 제시하더라도 실제 운영과 지속관리는 기초자치단체의 지역자원과 주민 참여 기반에 따라 조정될 수 있음을 보여준다.

### 5. 다른 지역에서의 적용 가능성

건강UP 사업을 다른 지역에서 적용하기 위해 부산의 마을건강센터와 동일한 시설을 새롭게 설치해야 하는 것은 아니다. 마을건강센터는 건강UP 사업에서 소생활권 건강관리 거점의 기능을 담당하지만, 다른 지역에서는 보건소, 보건지소, 건강생활지원센터, 보건진료소, 또는 지역주민이 지속적으로 접근할 수 있는 기존 공공시설이 유사한 역할을 담당할 수 있다.

다른 지역에서 적용할 때 중요한 것은 마을건강센터라는 명칭이나 시설을 그대로 따라 하는 것이 아니라, 소생활권 거점, 표준화된 노쇠선별, 다영역 중재, 프로그램 종료 후 주민활동과 재평가라는 핵심 기능을 확보하는 것이다. 여기에 전문인력과 교육받은 주민 인력의 역할을 구분하고, 지역의 기존 보건‧의료‧복지자원과 연결하는 운영체계를 마련할 필요가 있다.

기존 주민 조직이 잘 발달한 지역은 해운대구와 같이 기존 조직을 활용할 수 있으며, 그렇지 않은 지역은 노쇠예방관리교실 수료자를 중심으로 소규모 자조모임부터 단계적으로 형성할 수 있다. 노인일자리사업과 연계할 여건이 없는 지역에서는 보건소의 건강리더, 자원봉사자 또는 지역 내 주민활동가를 활용할 수 있다. 중요한 것은 부산의 조직명과 운영방식을 똑같이 만드는 것이 아니라 지역에서 이용 가능한 인력과 공간을 노쇠예방기능에 맞게 재구성하는 것이다.

이러한 관점은 지역사회 통합돌봄 정책과도 연결될 수 있다. 노쇠는 의료적 질환이 발생한 이후에만 관리해야 하는 문제가 아니라 장애와 장기요양으로 진행되기 이전부터 지역사회에서 발견하고 관리할 필요가 있다. 향후 노쇠예방사업이 보건사업과 의료‧복지‧돌봄서비스 사이의 연결점으로 발전한다면 건강한 노인에서 전노쇠, 노쇠 및 돌봄 필요 단계로 이어지는 기능 변화에 따라 보다 연속적인 지원이 가능할 것이다.

### 6. 사업평가의 한계와 향후 과제

본 글에서 제시한 참여자의 사전‧사후 결과는 지역사회 사업 수행 과정에서 축적된 자료를 활용한 것으로 해석에 주의가 필요하다. 대조군이 없는 단일군 분석이므로 프로그램 참여 전후의 차이를 건강UP 사업의 인과적 효과로 단정할 수 없다. 또한 참여자의 대부분이 여성으로 구성되어 있어 전체 지역사회 노인에게 결과를 일반화하는 데 한계가 있다.

지역사회 현장에서 수집된 체성분 자료는 측정조건과 입력자료의 표준화가 충분하지 않았으므로 결과 해석에 주의가 필요하다. 향후에는 자료입력 검증과 측정조건 표준화를 강화할 필요가 있다.

사업의 성과지표도 확장할 필요가 있다. 현재의 악력, 의자 일어서기, 우울 및 사회적 고립감과 같은 단기 지표뿐 아니라 신체활동량, 낙상, 의료이용, 장기요양 진입 및 일상생활기능의 장기적 변화 등을 추적한다면 지역사회 노쇠예방사업의 정책적 효과를 보다 객관적으로 평가할 수 있을 것이다.

건강UP 사업의 지속가능성을 위해서는 참여자 수의 확대뿐 아니라 사업을 수행하는 인력의 역량과 역할을 표준화할 필요가 있다. 마을간호사, 마을건강활동가, 영양사와 운동전문인력, 하하건강파트너가 담당할 수 있는 업무범위를 구체화하고, 주민 인력이 전문적인 판단을 요구하는 영역을 담당하지 않도록 교육과 감독체계를 마련하는 것이 중요하다.

부산광역시 건강UP 사업은 마을건강센터라고 하는 보건소의 소생활권 건강관리 기반시설을 활용하여 지역주민의 노쇠 위험을 조기에 발견하고, 전노쇠군을 중심으로 다영역 중재를 제공하며, 프로그램 이후에도 주민의 건강생활실천이 지속될 수 있도록 건강동아리와 주민 참여 활동을 연계한 지역사회 노쇠예방사업이다.

해운대구 운영 경험은 이러한 모형이 실제 지역사회에서 적용 가능하며, 프로그램 종료 이후 건강동아리와 주민 참여 활동으로 지속 연계할 수 있음을 보여주었다. 특히 건강동아리와 건강지킴이단은 주민의 자발적 건강활동을 촉진하고, 하하건강파트너는 노쇠검사, 프로그램 운영과 추적관리의 실무를 보완하였다. 이는 노인이 건강서비스의 수혜자에 머무르지 않고 동년배의 건강생활실천을 지원하는 주민 인력으로 참여할 가능성을 보여주었다.

건강UP 사업과 해운대구의 운영 경험에서 알 수 있는 핵심은 특정 프로그램이나 시설의 형태 자체보다 지역사회에서 노쇠예방사업이 지속될 수 있는 구조를 만드는 데 있다. 소생활권 건강관리 거점을 중심으로 대상자를 발굴하고 노쇠 여부를 선별한 후 다영역 중재를 제공하며, 프로그램 수료자를 자조모임과 주민 조직으로 연결하고, 전문인력과 교육받은 주민 인력이 역할을 분담하여 지속적인 건강활동과 재평가를 지원하는 구조가 그 핵심이다.

따라서 부산의 마을건강센터와 같은 조직이 없는 타 지역에서도 보건소, 건강생활지원센터, 보건진료소 등 기존 지역자원을 활용하여 이러한 핵심 기능을 지역 여건에 맞게 구성한다면 유사한 노쇠예방사업의 적용이 가능할 것으로 생각된다. 부산광역시 건강UP 사업은 향후 국가 노쇠예방정책과 지역사회 통합돌봄 정책의 발전에 참고모델로 활용될 수 있을 것이다.

## Figures and Tables

**표 1:** 마을건강센터 운영인력과 건강UP 사업에서의 역할

구분	마을건강센터의 기본 역할	건강UP 사업에서의 역할
담당자(공무원)	사업 기획·운영, 보건소 및 지역기관 연계, 주민공동체 지원	사업 총괄 및 운영 조정, 참여인력·지역자원 연계
마을간호사	건강상담, 만성질환 관리·교육, 건강위험군 관리	노쇠 관련 검사 및 신체측정, 결과상담 및 건강관리
마을건강활동가	주민 발굴·조직화, 주민교육, 건강리더 및 건강소모임 지원	대상자 발굴, 건강UP 교실 운영, 건강동아리 등 주민활동 지원

**표 2:** 건강UP 사업과 연계된 주민 인력 및 주민 조직

구분	하하건강파트너	건강동아리	건강지킴이단
성격	교육받은 주민 운영인력	건강UP 수료자 중심 자조모임	기존 주민 건강조직
참여방식	노인일자리사업 연계‧교육‧배치	수료자 중 희망자 중심 구성	기존 마을건강사업을 통해 구성
주요 역할	노쇠검사‧프로그램 운영‧재검 및 소모임 활동 지원	운동‧영양 등 건강생활실천 지속	주민 건강활동, 건강취약주민 발굴‧지원 및 지역자원 연계
건강UP과의 관계	사업 운영 및 지속관리 지원	프로그램 종료 후 지속관리	기존 주민 기반을 활용한 지역사회 연계

**표 3:** 건강UP 교실의 10주 프로그램 구성

회차	영역	주제	내용	담당
1	사업안내 및 소개	입학식	마음 열기: 자기소개, 참가 동기 말하기 사업안내, 프로그램 소개 노쇠란 무엇일까요?	마을건강활동가, 담당자, 마을간호사, 하하건강파트너
	운동	근력 강화 운동법 배우기(난이도 하)	기본동작 - 혼자 할 수 있는 운동 - 한 동작씩 배우고 실습하기 동작 시 주의사항 알기 교육자료 및 실천점검표 사용방법 안내	마을건강활동가, 하하건강파트너
2	영양	근력 유지를 위한 식이	기본동작 함께 해보기(복습) 제대로 알고 제대로 먹자(단백질 음료 소개) 실천점검표 사용방법 안내	보건소 영양사, 하하건강파트너
3	인지+운동	뇌를 젊게 만들기	기본동작 함께 하기(반복 시행) 인지복합게임/운동	마을건강활동가, 하하건강파트너
4	운동	근력 강화 운동법 배우기(난이도 중)	추가동작 - 다른 사람과 함께 하는 운동 - 한 동작씩 배우고 실습하기 동작 시 주의사항 알기	마을건강활동가, 하하건강파트너
5	공동체	공동체 일원으로 살기	추가동작 함께 해보기(복습) 공동체 교육(40분)	마을건강활동가, 하하건강파트너
6	사회적 활동	마을나들이(활동)	기본/추가동작 함께 하기(반복 시행) 마을 탐색 - 운동하기 좋은 장소 알기 - 자기만의 걷기 코스 개발 - 내게 적절한 걷기 운동 강도 알기	마을건강활동가, 마을간호사, 하하건강파트너
7	영양	근력 유지를 위한 식이	기본/추가동작 함께 하기(반복 시행) 올바른 식습관 실천 여부 점검 함께 건강식 만들기	마을건강활동가, 마을간호사, 하하건강파트너
8	인지+운동	뇌를 젊게 만들기	기본/추가동작 함께 하기(반복 시행) 인지복합게임/운동	마을건강활동가, 하하건강파트너
9	운동	호흡근을 강화하는 운동법 배우기	기본/추가동작 함께 하기(반복 시행) 호흡근 강화 운동 한 동작씩 배우고 실습하기 - 소음 측정기, 노래부르기, 빨대 등 활용	마을건강활동가, 하하건강파트너
10	사회적 활동	소모임 활동을 위한 워크숍	기본/추가동작 함께 하기(반복 시행) 호흡근 강화 운동 함께 하기(복습) 추후 건강소모임 활동 계획 의논	마을건강활동가, 하하건강파트너
	마무리	수료식	보건소장 인사, 수료증 증정 성과 발표 및 포상	담당자, 보건소관계자

**표 4:** 건강UP 교실 참여자의 사전‧사후 지표 변화

지표	분석(n)	사전 평균	사후 평균	평균 변화량	p-value
사지근육량(kg)	137	18.45±4.41	17.47±2.34	–0.97±3.84	0.028
악력(kg)	139	20.72±5.23	21.62±5.31	0.90±3.37	<0.001
의자 일어서기(초)	139	10.32±3.50	9.07±3.28	–1.25±3.17	<0.001
우울(점)	139	4.04±3.59	2.83±3.31	–1.20±2.79	<0.001
사회적 고립감(점)	139	4.25±1.58	3.88±1.41	–0.37±1.26	<0.001

변화량은 사후-사전으로 산출하고, 데이터는 평균±표준편차로 나타냄. 의자 일어서기, 우울 및 사회적 고립감은 점수 또는 시간이 감소할수록 개선을 의미. p-value는 양측 Wilcoxon 부호순위검정 결과.
